# Structured reporting of B-mode, color Doppler, and CEUS in testicular tumor assessment: a reader study with urologist ratings

**DOI:** 10.1186/s12894-026-02159-5

**Published:** 2026-04-24

**Authors:** Moritz Ludwig Schnitzer, Gloria Biechele, Clara Eckermann, Felix L. Herr, Christian Dascalescu, Maurice Heimer, Vincent Schwarze, Julian Marcon, Maximilian Lenhart, Frank Waldbillig, Fabian Baier, Dirk-Andre Clevert, Matthias Frank Frölich, Johannes Rübenthaler, Thomas Geyer

**Affiliations:** 1 Department of Radiology, University Hospital Munich, Ludwig Maximilian University of Munich, Munich, Germany; 2 Department of Urology, University Hospital Munich, Ludwig Maximilian University of Munich, Munich, Germany; 3 Department of Urology & Urosurgery, University Medical Center Mannheim, Heidelberg University, Mannheim, Germany; 4 Department for Radiotherapy, University Hospital Regensburg, Regensburg, Germany; 5 Department of Radiology and Nuclear Medicine, University Medical Center Mannheim, Heidelberg University, Mannheim, Germany

**Keywords:** Structured reporting, CEUS, Free-text reports, Testicular lesions

## Abstract

**Purpose:**

Structured reporting (SR) offers standardized radiological documentation, enhancing clarity and reproducibility. However, its role in contrast-enhanced ultrasound (CEUS) for testicular tumors remains underexplored. This study evaluates urologist-perceived clarity, completeness, and clinical usefulness of SR compared to free-text reporting (FTR).

**Methods and materials:**

In this retrospective, single-center study, 65 male patients with suspected testicular tumors underwent CEUS at LMU University Hospital. Reports were initially documented as FTRs by an experienced radiologist and later converted into SRs using Smart Reporting software. Four board-certified urologists independently assessed both formats using a structured questionnaire. Completeness, readability, trust, and impact on clinical decision-making were evaluated. Statistical analysis included McNemar’s test and the Wilcoxon signed-rank test, with α = 0.05.

**Results:**

SRs significantly improved readability (97.3% vs. 10.0%, *p* < 0.001) and information extraction (98.8% vs. 91.9%, *p* < 0.001). However, completeness (56.9% vs. 60.8%, *p* = 0.427) and clinical decision support (85.7% vs. 84.9%, *p* = 0.152) were comparable. Trust in SRs was lower than in FTRs (4.92 vs. 5.22, *p* < 0.001), likely due to missing diagnostic parameters and retrospective SR generation.

**Conclusions:**

SR was associated with improved reporting clarity and consistency but did not outperform FTR in completeness or clinical decision-making. Interdisciplinary collaboration in template development and the integration of classification systems could improve SR’s diagnostic value. Future prospective, multicenter studies should assess real-time SR implementation and its potential impact on reporting quality, communication, and outcome-based endpoints in prospective settings.

**Clinical relevance/application:**

Structured reporting in multiparametric testicular ultrasound including CEUS improved perceived readability and facilitated information access for referring clinicians. However, SR showed no clear advantage over free-text reporting regarding completeness or clinical decision-making. The lower clinician trust in SR highlights the need for clinically tailored templates developed in interdisciplinary collaboration. The broader clinical value of SR in testicular imaging should be confirmed in prospective real-time studies incorporating outcome-based and workflow-related endpoints.

## Background

Testicular tumors encompass a broad spectrum of benign and malignant neoplasms and represent the most common malignancy in young adult men [1–4]. Ultrasound is the primary diagnostic modality, and contrast-enhanced ultrasound (CEUS) has further improved lesion characterization by providing high–temporal resolution assessment of enhancement patterns and microvascularity. CEUS has therefore become an important complementary tool, although its diagnostic performance remains dependent on standardized contrast administration, scanner-specific presets, and operator experience, which can limit reproducibility across institutions [5–11].

Parallel to advances in imaging, structured reporting (SR) has gained increasing attention as an alternative to conventional free-text reporting (FTR). SR introduces predefined sections, standardized terminology, and consistent organization, aiming to improve clarity, completeness, and communication between radiologists and referring clinicians. In several radiological subspecialties including breast, prostate, thyroid, and liver imaging SR has been associated with improved report quality, greater inter-reader consistency, and more efficient information retrieval for clinicians [12–18]. These systems have also shown that structured templates can support diagnostic decision-making by reducing ambiguity and ensuring that key features are consistently documented.

However, the implementation of SR in routine clinical practice remains variable. Reported barriers include concerns about reduced flexibility, potential increases in reporting time, interruptions to established workflows, and uncertainty regarding how rigid templates fit into complex or atypical clinical scenarios. Successful SR adoption therefore depends on balancing standardization with usability and aligning template content with the needs of referring clinicians [19, 20].

In contrast to other organ systems, testicular ultrasound lacks an established structured reporting framework, despite the modality’s central role in the assessment of scrotal pathology. Given that CEUS provides dynamic information on lesion vascularity, a structured format may help present multiparametric findings from B-mode, Color Doppler, and CEUS in a clear and clinically meaningful way [6, 7]. Yet the extent to which SR improves clinical interpretability or communication in testicular imaging has not been systematically evaluated.

This study therefore aims to compare SR and FTR for multiparametric testicular ultrasound, including CEUS, in terms of clarity, completeness, and clinical usefulness. By assessing how urologists interpret, trust, and extract information from both report formats, this analysis seeks to determine whether structured reporting can improve interdisciplinary communication and potentially support more consistent diagnostic decision-making in testicular tumor evaluation.

## Methods

### Study design

This single-center study analyzed the benefits of SR compared to FTR based on radiological findings. Patient selection was based on predefined inclusion and exclusion criteria. The study included male patients who underwent CEUS at LMU University Hospital between 2011 and 2021. Patient age was available for all cases and was added as a descriptive characteristic (mean 47.1 years, range 28–90 years). The mean tumor diameter was 13.2 mm, ranging from 4 to 38 mm. All patients presented with a suspected solid testicular tumor based on medical history and clinical examination, which was further evaluated through ultrasound imaging. Patients of all age groups were included, and pre-existing conditions were not considered exclusion criteria.

From an initial cohort of 372 patients who underwent CEUS of the testes between 2011 and 2021, 237 examinations were excluded because the indications were unrelated to solid testicular tumors (e.g., follow-up examinations, infertility, trauma, or inflammation). This resulted in 135 eligible patients with suspected malignant testicular lesions. From this cohort, 65 cases were randomly selected for detailed analysis. No further patient-level exclusions were applied within this eligible group. All CEUS examinations showing solid intratesticular lesions during the study period were included, regardless of whether the lesion was discovered due to symptoms (e.g., swelling, pain, palpable mass) or identified incidentally during unrelated examinations. This approach reflects the real-world diagnostic spectrum and ensures representativeness of the cohort. The sample size was determined based on previous studies evaluating the implementation of SR across various clinical conditions.

This retrospective single-center study was approved by the institutional review board (Ethics Committee, Medical Faculty, Ludwig Maximilian University (LMU) Munich; 17–087; date of approval: 14 March 2017). Informed consent was waived. All data were collected in accordance with the principles of the Declaration of Helsinki/Edinburgh 2022.

### Data collection and acquisition

#### Image acquisition

For the morphological evaluation of testicular lesions, conventional B-mode ultrasound and color Doppler imaging were performed, as these are standard diagnostic modalities for testicular pathology. In addition, CEUS was utilized to further refine the diagnosis. All examinations were performed using a high-frequency linear transducer (4–18 MHz), which is standard for scrotal imaging.

All examinations were conducted by a radiologist with extensive clinical experience and specialized expertise in CEUS, holding an EFSUMB Level 3 qualification.

#### Generation of free-text reports

All examinations were performed by a single radiologist with extensive clinical experience. B-mode ultrasound was used to assess lesion size, shape, and echogenicity, while color Doppler imaging evaluated lesion vascularization. CEUS was employed to visualize contrast agent uptake within the lesion, allowing for the observation of arterial-phase wash-in and venous-phase wash-out dynamics.

High-end ultrasound systems equipped with CEUS protocols were utilized for imaging, including the Samsung RS80 and RS85 (Seoul, Korea) and the Philips EPIQ 7 (Seattle, WA, USA). The examinations were conducted using a low mechanical index (< 0.2). The contrast agent used in this study was SonoVue^®^ (Bracco, Milan, Italy). Patients received an intravenous dose of 1.6–2.4 mL of SonoVue^®^, followed by a flush of 5–10 mL of sterile 0.9% sodium chloride solution. No adverse effects were reported during or after the examinations.

Findings were documented as free-text reports using speech recognition software (Philips Speech Magic Build 6.1, SP1543, 7/2007, Philips Speech Recognition Systems GmbH, Vienna, Austria) and were stored in both the patient records and the radiology information system (PACS/RIS: Visage^®^ Imaging, Visage 7 Enterprise Imaging Platform, Pro Medicus Limited, Richmond, Australia). 

#### Generation of structured reports

Structured reports were retrospectively generated based on the free-text reports documented in the patient records using the Smart Radiology online software (Smart Reporting GmbH, Munich, Germany; http://www.smart-reporting.com). The SRs were reconstructed solely from the information provided in the original free-text reports. The original ultrasound images were not re-evaluated during SR creation to avoid retrospective reinterpretation and to ensure that both reporting formats were based on identical source information. Smart Reporting allows for the customization of reporting templates tailored to specific clinical applications. The tool enables modality-specific customization of templates and ensures consistent terminology. Its integration in this study allowed the reconstruction of structured reports from existing free-text documentation while maintaining uniformity across all cases. These included lesion localization, size, echogenicity, color Doppler findings, contrast enhancement, and tissue stiffness. The structured report template used in this study reflects the standard workflow in our department during the study period. Parameters such as calcifications and vascular architecture were not part of the original template and were only added as incidental findings. Clinical information, such as medical history or prior surgeries, could be recorded in designated free-text fields. Additionally, suspected diagnoses or other notable findings were documented in these fields.

**Fig. 1:**
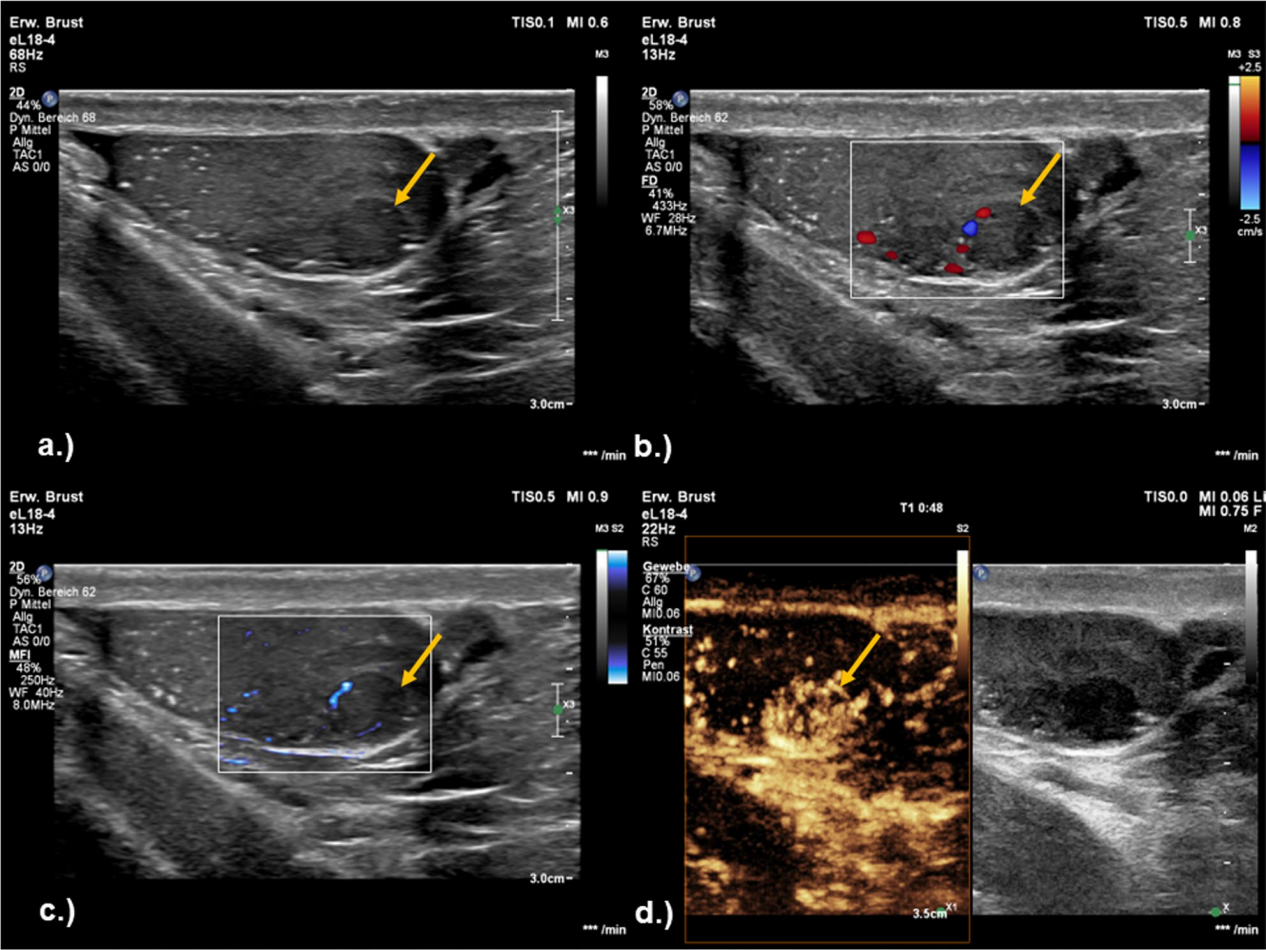
**a** Check-up right testis of a 51-year-old male patient in the follow up after Orchiectomy left due a Seminoma. He presented with a hypoechoic testicular lesion (yellow arrow). A high-frequency (4–18 MHz) linear probe was used for ultrasound examination. The diameter of the lesion is approximately 7 mm. **b** In color Doppler examination the lesion has slightly increased peripheral vessels (yellow arrow). **c** Micro Flow Imaging (MFI) detects peripheral slow and weak blood flow in the tumor tissue (yellow arrow). **d** After contrast application the lesion shows a moderate homogenous contrast uptake (yellow arrow)

**Fig. 2:**
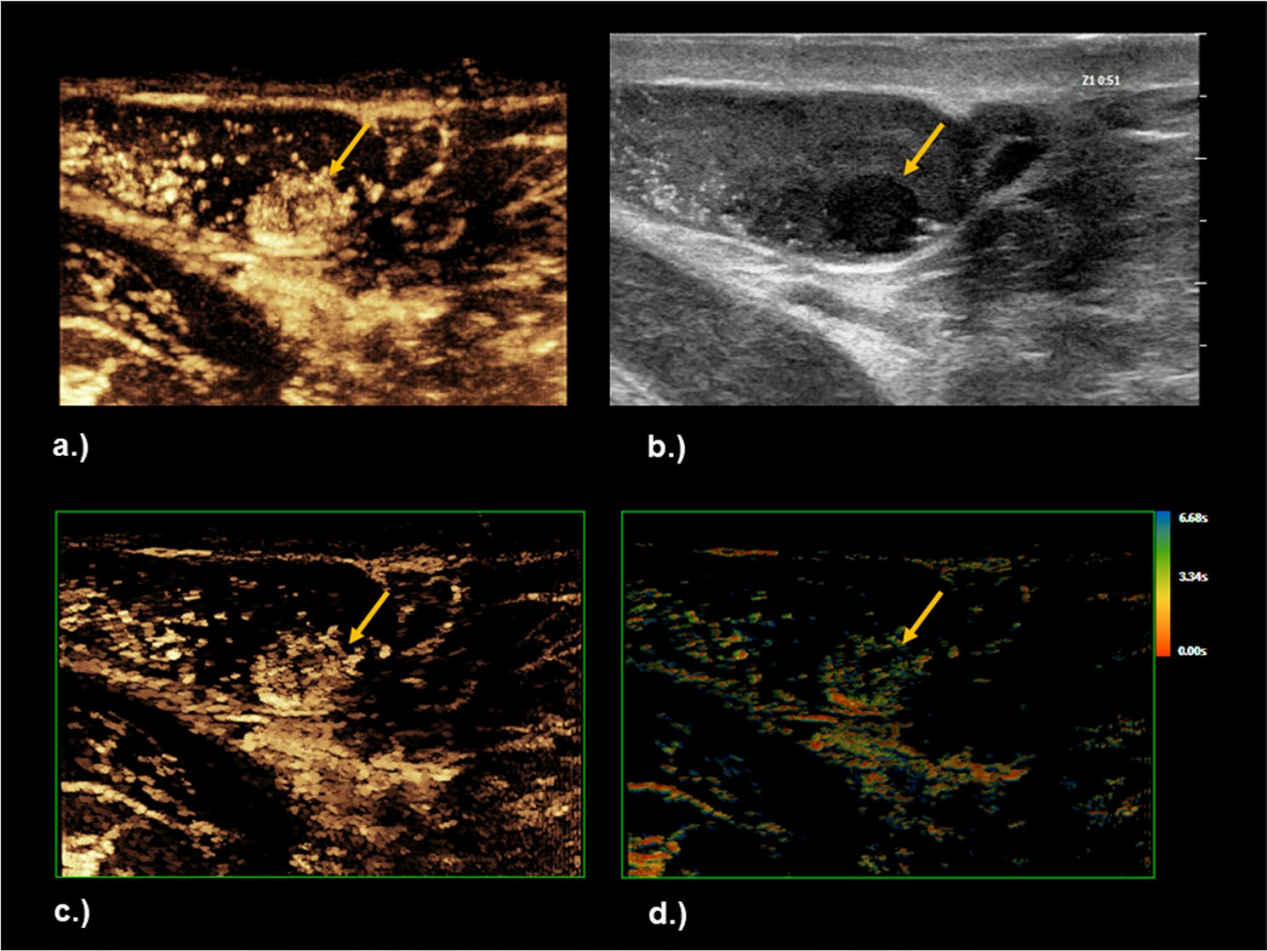
Same patient as Figure 1. After contrast application the lesion shows a moderate homogenous contrast uptake in comparison to the surrounding tissue (green arrows). In the Super Resolution CEUS Micro-Vascular Imaging (MVI SR) technique, tiny vessels could be depicted in higher resolution in comparison to the conventional CEUS setting. TOA SR post-processing techniques offers significantly enhanced visualization of the microvascular architecture, including feeding vessels, and a clear and concise way to evaluate and document the peak contrast enhancement and temporal arterial filling patterns. The lesion was confirmed as a seminomatous germ cell tumor on final histopathology. **a** Conventional CEUS examination shortly afterwards Figure 1d. **b** Grey scale examination (low MI). **c** Super Resolution CEUS Micro-Vascular Imaging (MVI SR) technique. **d** Time of Arrival Super Resolution (TOA SR) parametric mapping

**Fig. 3:**
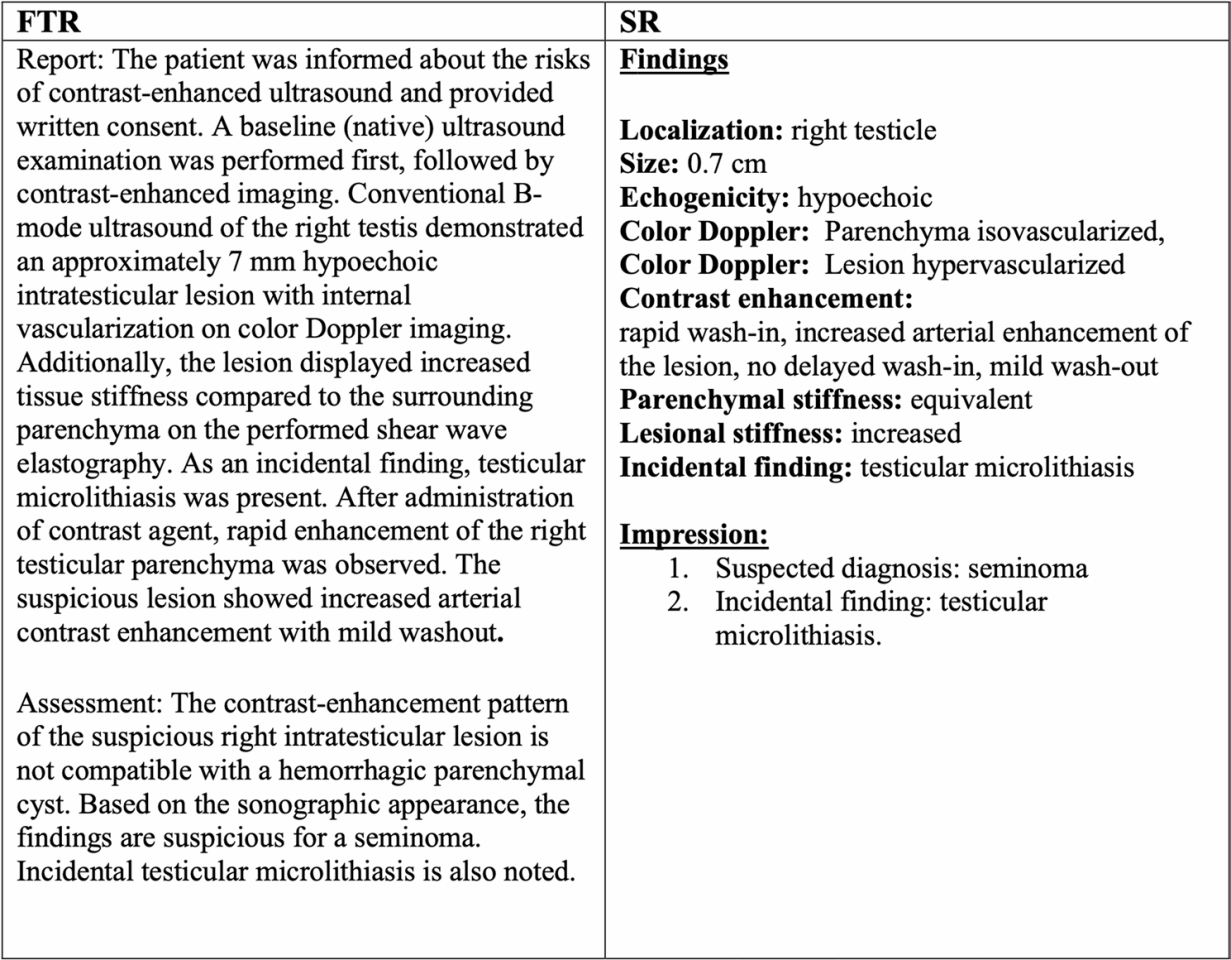
FTR and corresponding SR for the patient case

The platform incorporated predefined descriptors for each diagnostic feature, presented as concise keywords or short semantic phrases. The structured reporting process was facilitated by decision trees and drop-down menus, enabling the selection of appropriate descriptors for each lesion. Once all relevant descriptions were chosen and any necessary free-text information was added, Smart Reporting automatically generated a standardized, structured report containing all essential diagnostic details in a uniform format. An example case of a patient with a seminoma in multiparametric ultrasound including CEUS with a corresponding SR and a FTR can be seen in Figs. 1, 2 and 3. 

#### Evaluation of reports

To assess the quality of the two reporting formats and their potential impact on interdisciplinary communication between radiology and urology, SR and FTR for each of the 65 patient cases were provided to two urologists from the University Hospital of LMU Munich as well as two urologists from the University Hospital Mannheim. The reports were anonymized and randomly assigned to the urologists for evaluation. Each of the four urologists evaluated all 65 cases in both report formats, resulting in a maximum of 260 ratings per category. One response to the item on confirmation of tumor suspicion was left unanswered, yielding 259 valid ratings for this analysis. Missing items were treated as invalid according to the predefined evaluation rules. The urologists only received the written reports without access to the ultrasound images. To mitigate recall bias, all reports were anonymized and presented to the urologists in a fully randomized order. Because each evaluator reviewed all 130 reports, a washout period was not required, and paired reports were unlikely to be recognized.

**Fig. 4:**
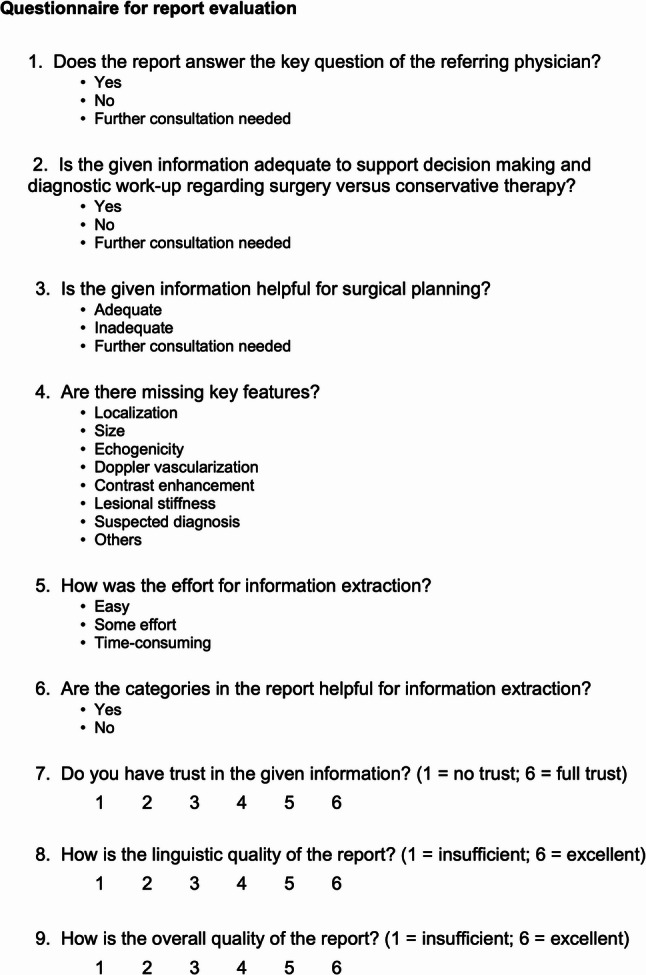
Questionnaire for the evaluation of FTR and SR evaluated by four board-certified urologists

To document the urologists’ assessments, a standardized questionnaire was developed (Fig. 4). The questionnaire was developed based on published literature on structured reporting, diagnostic confidence, and interdisciplinary communication [21–25]. Items were adapted to the context of testicular imaging and formulated jointly by two radiologists and two urologists to ensure clinical relevance. The draft questionnaire underwent expert review for face validity and was pilot-tested on two urologists not participating in the main study to confirm clarity and usability. Minor wording adjustments were made accordingly. The questionnaire consisted of nine questions, which the urologists were invited to complete. A 6-point Likert scale was used to avoid a neutral midpoint and to reduce central tendency bias. This forced-choice format encourages respondents to indicate a direction of preference and has been recommended in settings with small participant numbers to enhance discriminatory capacity. 

### Statistical methods

The evaluations provided by the urologists were systematically organized using the statistical software SPSS (IBM SPSS Statistics Version 29, Armonk, New York, NY, USA). The data were structured according to both individual questions and the respective evaluators (“Urologist 1 to 4”).

Subsequently, the data were analyzed for statistical significance using various test scenarios. The McNemar test was applied to compare binomial data, assessing the evaluations of the reporting formats. Additionally, the Wilcoxon signed-rank test was employed to analyze paired data by assessing deviations. This test determined whether the SR format led to consistency, improvement, or deterioration compared to FTR. The results were categorized into ranks (positive, equal, and negative ranks) and statistically evaluated accordingly. The significance level was set at α = 0.05.

## Results

### Confirmation of tumor suspicion

The ability to confirm tumor suspicion based on CEUS findings was reported in 88.8% of cases using the SR format and 85.7% with the FTR format (*p* = 0.060). In 9 out of 259 SR cases and 8 out of 259 FTR cases, the diagnosis remained inconclusive. Further investigations were necessary to substantiate tumor suspicion in these cases, including correlation with serum tumor markers, follow-up imaging, and, depending on the clinical scenario, surgical exploration with histopathological confirmation. For this item, one response was missing, resulting in 259 valid evaluations for SRs and 259 for FTRs.

### Clinical decision-making

Both report formats contributed comparably to clinical decision-making. The FTR format supported decision-making in 84.9% of cases, while the SR format did so in 85.7% (*p* = 0.152).

Similarly, for surgical planning, the SR format was deemed sufficient in 61.4% of cases, compared to 63.3% for the FTR format (*p* = 0.249). One urologist consistently marked all reports as insufficient for surgical planning, citing the necessity of additional tests (e.g., serum tumor markers). When excluding this urologist’s ratings, SR reports were considered sufficient in 81.0% of cases and FTR reports in 83.1% (*p* = 0.368). 

### Completeness of reports

The perceived completeness of SR reports was comparable to that of FTR reports (*p* = 0.427). SRs contained similar diagnostic details, including lesion localization, size, echogenicity, Doppler vascularization, contrast enhancement, tissue stiffness, and diagnostic suspicion. Missing elements were reported in 10.8% of FTRs and 9.6% of SRs.

Excluding one radiologist who consistently marked reports as incomplete due to the absence of additional clinical parameters (e.g., patient age, contralateral testicular size, tumor percentage in the affected testis), completeness ratings improved: 84.1% of FTRs and 84.6% of SRs lacked none or only one diagnostic element (*p* = 0.365).

### Readability and structure

SRs were significantly more readable than FTRs. In 97.3% of cases, SRs were rated as easy to read, whereas FTRs were deemed “some effort” (69.5%) or “very time-consuming” (20.5%) (*p* < 0.001).

The structured format and predefined categories in SRs were also rated as highly effective for information extraction (98.8% vs. 91.9% for FTRs, *p* < 0.001).

### Trust in reports

Urologists reported higher trust in FTRs (mean score: 5.22 ± 0.84) than in SRs (4.92 ± 1.22) (*p* < 0.001). While 85.7% of FTRs received high or full trust ratings, only 66.8% of SRs did. Notably, three SRs received no trust at all.

### Linguistic quality

The linguistic quality of SRs was slightly better than that of FTRs (mean score: 5.18 ± 0.93 vs. 5.07 ± 0.97), though the difference was not statistically significant (*p* = 0.089). SRs were rated as “outstanding” in 47.5% of cases, compared to 39.0% for FTRs. 

### Overall report quality

The overall report quality was rated slightly lower for SRs (mean: 4.94 ± 1.12) than for FTRs (5.05 ± 0.85) (*p* = 0.065). While SRs were rated as “outstanding” in 40.2% of cases (vs. 31.3% for FTRs), they also had a higher proportion of ratings ≤ 4 (33.2% vs. 19.3%).

Ratings differed between the two participating academic centers. Urologists from LMU Munich rated SRs more favorably (5.82 and 5.63 vs. 5.55 and 5.38 for FTRs), whereas urologists from Mannheim rated FTRs slightly higher (4.72 and 4.55 vs. 3.97 and 4.32 for SRs). The institutional attribution is provided solely to illustrate inter-rater variability and does not imply geographic relevance. An overview can be seen in Table 1.

**Table 1:** Rater-stratified scores for overall report quality (Likert 1–6): Mean ratings, standard deviations, and ranges for each of the four urologists are shown separately for FTR and SR. The table illustrates inter-rater variability as well as the consistent tendency of LMU raters to assign higher quality scores compared with raters from Mannheim

Rater	Format	*n*	Mean	SD	Range (min - max)
Urologist 1 (LMU)	SR	65	5.82	0.464	4–6
Urologist 1 (LMU)	FTR	65	5.55	0.587	4–6
Urologist 2 (LMU)	SR	65	5.63	0.627	3–6
Urologist 2 (LMU)	FTR	65	5.38	0.860	2–6
Urologist 3 (Mannheim)	SR	64	3.97	0.642	2–5
Urologist 3 (Mannheim)	FTR	64	4.72	0.603	3–6
Urologist 4 (Mannheim)	SR	65	4.32	1.213	1–6
Urologist 4 (Mannheim)	FTR	65	4.55	0.848	2–6

### Qualitative feedback

Urologists at LMU Munich provided no additional comments. In contrast, those from Mannheim University Hospital noted the absence of key parameters in both report formats, including patient age, clinical history, testicular volume, tumor percentage within the testis, and whether a prosthesis was considered. One urologist criticized the reliance on imaging alone for surgical decision-making, advocating for additional biomarker assessment.

One urologist suggested that future structured reports could incorporate a standardized risk-stratification approach (e.g., a BI-RADS/TI-RADS–like classification) including a defined lexicon and an explicit malignancy-suspicion scale. A summarized overview of all questions can be seen in Table 2.

**Table 2:** Questionnaire results. Values are percentages of affirmative responses unless stated otherwise; Likert-scale items are reported as mean ± SD

Question	FTR	SR	*p*
Does the report answer the key question of the referring physician?	85.7%	88.8%	0.060
Is the given information adequate to support decision making and diagnostic workup regarding surgery versus conservative therapy?	84.9%	85.7%	0.152
Is the given information helpful for surgical planning?	63.3%	61.4%	0.249
Are there missing key features?	10.8%	9.6%	0.427
How was the effort for information extraction?	69.5% (“some effort”), 20.5% (“very time-consuming”)	97.3% (easy to read)	< 0.001
Are the categories in the report helpful for information extraction?	91.9%	98.8%	< 0.001
Do you have trust in the given information?	5.22 ± 0.84	4.92 ± 1.22	< 0.001
How is the linguistic quality of the report?	5.07 ± 0.97	5.18 ± 0.93	0.089
How is the overall quality of the report?	5.05 ± 0.85	4.94 ± 1.12	0.065

## Discussion

The findings of this study indicate that SRs were perceived as notably clearer and easier to read than traditional FTRs. Urologists rated 97.3% of SRs as easy to read, compared with only 10.0% of FTRs, and information extraction was significantly more efficient in the structured format (*p* < 0.001). Despite these advantages, SRs and FTRs performed similarly with respect to key clinical endpoints. Confirmation of a suspected testicular tumor was possible in 88.8% of SRs and 85.7% of FTRs, a difference that did not reach statistical significance (*p* = 0.060). Clinical decision-making was supported in 85.7% (SR) and 84.9% (FTR) of cases (*p* = 0.152), and surgical planning in 61.4% and 63.3%, respectively (*p* = 0.249). Completeness ratings were also comparable (56.9% SR vs. 60.8% FTR). Trust in the reports was slightly higher for FTRs (mean 5.22) than for SRs (mean 4.92, *p* < 0.001), an expected finding given that SRs were reconstructed retrospectively and therefore restricted to parameters consistently documented in the original free-text reports. The observed disparity between the markedly improved readability of SRs and their slightly lower trust ratings is closely related to this methodological constraint. Because SRs were generated exclusively from diagnostic elements reliably present in the original FTRs, the resulting template was necessarily concise and could not incorporate semiological features that were inconsistently documented, such as calcifications or detailed vascular architecture. This perceived diagnostic brevity, despite both formats containing identical clinical information, likely contributed to reduced confidence among some evaluators. These findings underscore that achieving higher diagnostic trust requires prospectively developed templates that capture a broader and clinically comprehensive set of parameters.

These results are consistent with evidence from other radiological subspecialties, where structured reporting systems such as BI-RADS, PI-RADS, LI-RADS, and TI-RADS have demonstrated clear advantages in clarity, standardization, and report completeness [18, 21, 26–29]. It should be emphasized that the SR template evaluated in this study was not designed as a risk-stratification framework comparable to TI-RADS or other ‘RADS’ systems. In particular, it does not provide a dedicated lexicon or an explicit malignancy-suspicion score; rather, it structures the documentation and presentation of multiparametric ultrasound findings. Developing a consensus-based lexicon and suspicion scale for testicular lesions remains an important objective for future prospective work. At the same time, these systems have highlighted challenges associated with template rigidity, increased reporting time, and the need for continuous interdisciplinary refinement [22–25, 30–32]. Similar dynamics appear relevant in testicular imaging: the structured format in our study provided improved organization and language consistency, but its diagnostic value was ultimately limited by the content available in the original free-text documentation. Because the SRs were created retrospectively, they could include only parameters that were reliably present in every FTR. Features such as calcifications, vascular architecture, or detailed morphologic descriptors were not documented consistently enough to be incorporated. This constraint likely contributed to the somewhat lower trust ratings for SRs, as the template inevitably appeared more concise and potentially less comprehensive, even though diagnostic content between SR and FTR was identical.

Despite these constraints, the urologists’ evaluations indicate that SR primarily improves the clarity and organization of radiological information. The structured presentation may facilitate orientation within the report and reduce ambiguity in interdisciplinary communication. Because reporting or reading times were not measured, conclusions regarding workflow efficiency cannot be drawn. These findings therefore suggest a communicative rather than outcome-related advantage of SR in the present reader-study setting.

Several limitations must be considered. The retrospective design limited the analysis to parameters documented in routine care, and image data were not re-evaluated during SR reconstruction to avoid retrospective reinterpretation. Some clinically relevant descriptors were inconsistently available in the original reports, which restricted the diagnostic breadth of the SR template. Although lesion size was documented for all cases, the study was not designed or powered to examine whether lesion dimensions influenced urologists’ interpretations or confidence, and no subgroup analyses were conducted. Efficiency metrics such as reporting time or time-to-information were not collected and should be incorporated into future work. Differences in evaluations between centers also suggest that institutional expectations and reporting cultures influence how SRs are perceived. Finally, the study relied on subjective assessments from four urologists, which may limit the generalizability of the findings. Importantly, although SR improved perceived readability and information extraction, the primary clinically oriented endpoints (confirmation of tumor suspicion, clinical decision-making, and surgical planning) were comparable between SR and FTR.

In conclusion, structured reporting may contribute to clearer and more consistent communication in testicular ultrasound, although its broader clinical impact remains to be demonstrated prospectively.

## Conclusions

In this reader study, structured reporting was associated with improved perceived readability and facilitated information extraction compared with free-text reporting, whereas clinically oriented endpoints such as confirmation of tumor suspicion, clinical decision-making, and surgical planning remained comparable between both formats. These results indicate that the primary benefit of structured reporting lies in clearer presentation and communication of imaging findings rather than measurable changes in clinical decision processes. Prospective studies incorporating standardized lexicons, risk-stratification elements, and objective workflow metrics are warranted before structured reporting can be considered for routine implementation in testicular ultrasound.

## Data Availability

The datasets used and/or analysed during the current study are available from the corresponding author on reasonable request. The data is located in controlled access data storage in LMU Munich.

## Abbreviations

GCTs: Germ cell tumors
NSGCTs: Non-seminomatous germ cell tumors
CEUS: Contrast-enhanced ultrasound
SR: Structured reporting
FTR: Free-text reports
LMU: Ludwig Maximilian University
